# Bacteriophages JEP7 and PBC2, which target foodborne pathogens, elicit cytokine responses in mammalian cells

**DOI:** 10.1007/s10068-025-02042-3

**Published:** 2025-11-26

**Authors:** Yewon Jung, Jinshil Kim, Ju-Hoon Lee, Sangryeol Ryu

**Affiliations:** 1 Department of Food and Animal Biotechnology, Research Institute of Agriculture and Life Sciences, Seoul National University, Seoul, 08826 Republic of Korea; 2 Department of Agricultural Biotechnology, Seoul National University, Seoul, 08826 Republic of Korea; 3 Center for Food Bioconvergence, Seoul National University, Seoul, 08826 Republic of Korea; 4 Department of Food Science & Biotechnology, and Carbohydrate Bioproduct Research Center, Sejong University, Seoul, 05006 Republic of Korea; 5 Present Address: Gene Expression and Regulation Section, Laboratory of Biochemistry and Genetics, National Institute of Diabetes and Digestive and Kidney Diseases, National Institutes of Health, Bethesda, MD 20892 USA

**Keywords:** Bacteriophages, Cytokine responses, Foodborne pathogens, Inflammation, Phage therapy

## Abstract

Phage therapy provides a potential approach to combat foodborne pathogens, yet the impact of phages on mammalian immune responses remains insufficiently explored. This study investigated the effects of ten phages on inflammatory cytokine production in RAW 264.7 macrophages and Caco-2 cells. *Escherichia coli* phage JEP7 stimulated TNF-α, IL-6, and IL-10, while *Bacillus cereus* phage PBC2 induced IL-10 in RAW 264.7 cells. JEP7 and PBC2 also differentially induced TNF-α, IL-6, and IL-10 expression in Caco-2 cells, reaching levels comparable to those triggered by lipopolysaccharide, demonstrating cytokine production across cell types. Comparative analysis of phage internalization, cytotoxicity, and virion glycosylation revealed no differences between cytokine-inducing and non-inducing phages, suggesting that these factors do not significantly impact inflammatory responses induced by JEP7 and PBC2. These findings demonstrate that phages might stimulate inflammatory responses via differential cytokine induction, although common factors affecting immune responses among the analyzed phages remain unidentified and warrant further investigation.

**Supplementary Information:**

The online version contains supplementary material available at 10.1007/s10068-025-02042-3.

## Introduction

The emergence of antimicrobial resistance has recently posed significant challenges to global public health (Gupta and Datta, 2019). With rising concerns that multi-drug resistant (MDR) infections could become the foremost cause of mortality worldwide, the effectiveness of traditional chemotherapy using antibiotics and other antimicrobial agents has significantly diminished (Venkatesan, 2021). Bacteriophages (phages) are viruses that infect and lyse bacterial host specifically (Baker et al., 2018). Phage therapy, which utilizes strictly lytic phages to treat patients unresponsive to conventional antibiotics, has gained attention as a promising alternative to antibiotic treatment against MDR infections (Hatfull et al., 2022). Numerous studies have evaluated the therapeutic potential of phages in mammalian cells and animal models to ensure their effective application in clinical practice (Bhandare et al., 2018). Hence, gaining a deeper understanding of the effects of phage on the mammalian immune system is crucial to minimizing the risk of unpredictable adverse effects in clinical practice. The efficacy of phage therapy relies not only on the ability of phages to target and eliminate the bacterial hosts but also on the immune response of the mammalian hosts. Several studies have revealed that phage administration in vivo may affect innate immune responses in mammalian hosts (Popescu et al., 2021). However, the resulting inflammatory response is typically confined to the sites of inflammation and varies depending on the specific phage used (Dufour et al., 2019; Zhang et al., 2018). Thus, deeper understanding of the interplay between phages and the mammalian immune system is essential to enhance the efficacy and safety of phage therapy.

The inflammatory response is a fundamental defense mechanism of the innate immune system, enabling rapid responses to external threats like invading pathogens and maintaining immune homeostasis (Bieghs and Trautwein, 2013). Inflammation is mediated by the production of inflammatory cytokines, which are small signaling proteins responsible for modulating various immune activities. Pathogen-associated molecular patterns (PAMPs) on microbes are detected by immune cells through Toll-like receptors (TLRs), which function as key pattern recognition receptors (Matsumoto et al., 2011). Tumor necrosis factor-alpha (TNF-α), a major pro-inflammatory cytokines, is crucial in initiating immune responses by promoting the recruitment and stimulation of immune cells at sites of infection or tissue damage (Gonzalez Caldito, 2023). TNF-α is produced at an early stage in the inflammatory process and plays a regulatory role in the production of other pro-inflammatory mediators, including interleukin-6 (IL-6) (Inoue et al., 2014). Excessive IL-6 production is linked to chronic inflammatory diseases, underscoring the importance of balanced regulation of this cytokine (Tu et al., 2023). Conversely, IL-10 acts as an important anti-inflammatory cytokine, inhibiting pro-inflammatory cytokine expression in macrophages and resolving inflammation (Fiorentino et al., 1991).

To harness the therapeutic potential of phages, various administration routes, such as oral, inhalational, and parenteral delivery, have been explored. Even when delivered locally to infected sites, phages can circulate systemically and potentially influence immune responses in both infected and uninfected cells (Ryan et al., 2011; Uyttebroek et al., 2022). While most previous studies have focused on phage-induced immune responses in cells stimulated with bacteria or LPS, their effects in the absence of such external stimuli remain largely unexplored. This highlights the importance of evaluating phage-induced immune responses under both conditions to ensure safe clinical application.

In this study, we investigated the effects of ten phages on the production of these critical cytokines (TNF-α, IL-6, and IL-10) in RAW 264.7 mouse macrophages and Caco-2 human epithelial cells to gain insights into how phages trigger the inflammatory responses in both immune and non-immune cells. To reflect the cellular responses that may occur upon exposure to phages targeting foodborne pathogens, RAW 264.7 and Caco-2 cell lines were used as *in vitro* systems to evaluate immune and epithelial responses, respectively. Among the ten phages, *Escherichia coli* phage JEP7 was found to induce the production of TNF-α, IL-6, and IL-10, while *Bacillus cereus* phage PBC2 triggered IL-10 production in RAW 264.7 cells. In Caco-2 human epithelial cells, both JEP7 and PBC2 stimulated the expression of TNF-α, IL-6, and IL-10 to levels comparable to those stimulated by LPS. A comparative analysis of phage internalization, cytotoxicity, and virion glycosylation revealed no differences between groups of the phages that can affect cytokine production and those that cannot, suggesting the need for further research on a broader range of phages to fully elucidate the mechanisms of mammalian immune response against phage.

## Materials and methods

### Bacterial strains and culture conditions

All bacterial strains used for phage propagation in this study are listed in Table S1. *Clostridium perfringens* isolate 2722 was cultured at 37 °C under anaerobic conditions in Brain heart infusion (BHI) broth and agar (Difco, MI, USA). *Staphylococcus aureus* isolate FMB-1 was grown in Tryptic soy broth (TSB) (Difco, MI, USA) and Tryptic soy agar (Difco, MI, USA) at 37 °C. All other bacterial strains were cultivated in Luria–Bertani (LB) medium (Difco, MI, USA) at 37 °C.

### Phage purification

The incubation time for phage propagation was adjusted according to the lysis activity of each phage. Following phage propagation, all ten phage lysates were precipitated with 10% (w/v) polyethylene glycol (PEG) 6000, separated using cesium chloride (CsCl) density gradient ultracentrifugation, and dialyzed in 1 L of sodium chloride-magnesium sulfate (SM) buffer (100 mM NaCl, 8 mM MgSO_4_·7H_2_O, and 50 mM Tris·HCl, pH 7.5) as previously described (Kim and Ryu, 2011).

To further remove residual contaminants such as LPS endotoxin, the four *E. coli* phages, including JEP5, JEP6, JEP7, and 6MG, were purified using the Endotrap HD columns (Lionex, Braunschweig, Germany) according to the manufacturer’s protocol. Endotoxin concentrations were determined using the Pierce™ LAL Chromogenic Endotoxin Quantitation Kit (Thermo Fisher Scientific, MA, USA). Additionally, *B. cereus* phage PBC2 was further purified using a Zeba Spin desalting column (Thermo Fisher Scientific, MA, USA) to remove residual salts, including MgCl_2_ and CaCl_2_ that were introduced during phage propagation, thereby preventing their potential influence on cytokine responses.

### Cell lines and culture conditions

Caco-2 (RRID: CVCL_0025, ATCC HTB-37) and RAW 264.7 (RRID: CVCL_0493, ATCC TIB-71) cells were obtained from the American Type Culture Collection (VA, USA). According to the provider, short tandem repeat (STR) authentication has been performed for Caco-2 cells, and both cell lines are reported to be free of mycoplasma and other microbial contamination. Cells were used within five passages and exhibited typical morphology and growth characteristics. Cells were cultured in Dulbecco’s modified eagle medium (DMEM; ATCC, VA, USA) containing 10% fetal bovine serum (FBS) (Invitrogen, CA, USA) and incubated at 37 °C under 5% CO_2_. Trypan blue staining was used to assess cell viability.

### RNA extraction and quantitative reverse transcription PCR (qRT-PCR)

To assess the effect of phage treatment on the mRNA expression of TNF-α, IL-6, and IL-10 in macrophages and epithelial cells, RAW 264.7 cells and Caco-2 cells were seeded in a 12-well plate at a density of 2 × 10^5^ cells per well and 10^5^ cells per well, respectively. After 24 h, 10^8^ or 10^9^ plaque-forming units (PFU) per mL of phages, LPS (1 μg/mL, ≥ 500 endotoxin units (EU)/mL) (Sigma-aldrich, MO, USA), and SM buffer were added to each well and incubated for 24 h. Phage concentrations of 10^8^–10^9^ PFU/mL were selected to represent upper-limit exposure conditions commonly used in cell culture experiments to assess immune responses induced by phages. Total RNA extraction was performed using the TRIzol reagent (Invitrogen, CA, USA) according to the manufacturer’s instructions. Briefly, the cells detached by TRIzol reagent were treated with 250 μL of chloroform with vigorous shaking to separate the aqueous phase containing RNA and the organic phase. After incubation at room temperature for 5 min, the samples were centrifuged at 8000 × g for 5 min. 400 μL of the aqueous phase was separated and treated with 550 μL of isopropanol. After centrifugation at 12000 × g for 30 min, 1 mL of 75% ethanol in DEPC-treated H_2_O was added to the samples. RNA was pelleted by centrifugation at 8000 × g for 5 min followed by air-drying. Samples were tre

ated with the Turbo DNA-free kit (Ambion, TX, USA) to eliminate the genomic DNA contamination. The cDNA was synthesized and then combined with 2 × iQ SYBR Green Supermix (Bio-Rad, CA, USA) and 0.3 μM of each primer in a total reaction volume of 20 μL. qRT-PCR was performed using the CFX Connect Real-Time PCR detection system (Bio-Rad, CA, USA) employing the primer sets listed in Table S2. Primers designed in this study were created using the NCBI Primer-BLAST software. The transcription level of glyceraldehyde-3-phosphate dehydrogenase (GAPDH) was used for normalization.

### Enzyme-linked immunosorbent assay (ELISA)

For the quantification of inflammatory cytokines in RAW 264.7 cells incubated with phages, we used the mouse TNF-α, IL-6 and IL-10 ELISA kits (Labis KOMA, Seoul, Korea) according to the manufacturer’s protocol. After the phages were incubated with RAW 264.7 cells for 24 h, the supernatants were used for the ELISA assay. Briefly, each well was washed 3 times with washing solution, followed by the addition of standards, blanks, and samples (in duplicate). After incubation for 2 h at room temperature, wells were aspirated and washed 4 times. Subsequently, detection antibody was added to each well and the p was incubated for 2 h. After another washing step, 100 μL of diluted Streptavidin-HRP was added, and the plate was incubated for 30 min. After washing the plate, TMB substrate was applied to initiate color development, and absorbance was measured at 450 nm using a microplate reader. A standard curve was generated by the absorbance of serially diluted standard proteins and corresponding known concentrations.

### Phage internalization assay

The phage internalization into mammalian cells was assessed using the method according to (Bichet et al., 2021). Briefly, the cells were grown in a 12-well plate with the same conditions used for RNA extraction. After 24 h incubation, phage preparations with different concentrations (10^8^ or 10^9^ PFU/mL) were added to each well and incubated for another 24 h. The cells were detached by adding 0.5 mL of trypsin and pelleted by centrifugation at 400 × g for 4 min. Cells were washed with ice-cold PBS and lysed with 0.5 mL lysis buffer (2% sodium deoxycholate, 10 mM Tris–HCl, 2 mM EDTA, pH 8.0). After a short vortexing for 10 s, the cells were incubated for 1 h at room temperature. The lysed cells were vortexed for 10 s, and the phage concentrations were quantified by titration. To account for extracellular phages, non-lysed controls were processed in parallel. The internalized phages were quantified by subtracting PFU from non-lysed samples from those of lysed samples.

### Cell cytotoxicity assay

The cytotoxicity of phages in RAW 264.7 cells was measured by lactate dehydrogenase (LDH) release assay. Briefly, cells were seeded in 96-well plates with a density of 2 × 10^4^ cells/well. Varying concentrations (10^8^ and 10^9^ PFU/mL) of the phages were inoculated into each well and incubated for 24 h. Subsequently, cells were centrifuged at 130 × g for 5 min to obtain the supernatant and cell cytotoxicity was measured using cytotoxicity detection kit (LDH) (Roche, Mannheim, Germany). The absorbance was measured at a wavelength of 495 nm with a SpectraMax i3 platform (Molecular Devices, CA, USA). The results were expressed as values relative to untreated cells.

### Sodium dodecyl sulfate–polyacrylamide gel electrophoresis (SDS-PAGE) and glycoprotein staining

Glycosylated proteins of the phages were examined using a Pierce™ Glycoprotein Staining Kit (Thermo Fisher Scientific, MA, USA) as previously described (Freeman et al., 2023). Briefly, 2 × 10^10^ PFU/mL of the phages were pelleted by centrifugation at 13000 × g for 30 min, resuspended in dithiothreitol (DTT), and treated with 2 μL of DNase I (1 mg/mL) to remove the DNA. Phage preparations were denatured and loaded into each well of 12% SDS polyacrylamide gel. Phage proteins were separated by electrophoresis at 120 V for 1 h and stained with either Coomassie blue or Glycoprotein stain.

### Statistical analysis

Statistical analyses were performed using GraphPad Prism version 5.01 (GraphPad Software, Inc., CA, USA). One-way ANOVA followed by Dunnett’s post hoc test was used for multiple group comparisons. Statistical comparisons between phage-treated groups and the positive control were performed using an unpaired Student’s *t*-test with Welch’s correction. *p* values < 0.05 were considered statistically significant.

## Results and discussion

### Selected phages induce cytokine production

In order to investigate the effects of phages on inflammatory cytokine expression in macrophages, we tested ten phages with different morphotypes, life cycles, and bacterial hosts (Table S1). Phage lysates often contain remnants of bacterial RNA, DNA, and other host-derived toxic components, which could trigger an immune response (Van Belleghem et al., 2017b). To remove these contaminants, all phage lysates were purified using CsCl density gradient ultracentrifugation, followed by dialysis (Reddy et al., 1988; Van Belleghem et al., 2017a). After purification, the phage preparations were incubated with RAW 264.7 cells for 24 h, and the expression levels of three inflammation-associated cytokines (TNF-α, IL-6, and IL-10) were assessed by qRT-PCR. Among the phages infecting Gram-negative bacteria, four *E. coli* phages including JEP5, JEP6, JEP7, and 6MG induced the expression of all three cytokines at levels comparable to those observed in LPS-stimulated cells (data not shown). In contrast, the remaining phages did not trigger cytokine expression. Since LPS from Gram-negative bacteria can mediate inflammatory responses in mammalian cells via TLR4 signaling (Gay et al., 2014), the four phages that triggered cytokine expression were subsequently purified using Endotrap HD columns to remove residual endotoxin that may have contaminated the phage preparations. Prior to endotoxin removal, the four phages contained varying concentrations of endotoxin, ranging from 12.5 to 957.8 EU/mL at a phage concentration of 10^9^ PFU/mL (Table S3). After purification, the endotoxin levels in all four phages were reduced to below the acceptable limit of 0.5 EU/mL, as specified by the United States Pharmacopeia (USP) guidelines (Hietala et al., 2019). The purified phages were subsequently tested for their ability to induce cytokine responses.

### JEP7 and PBC2 stimulated cytokine production in macrophages

In this study, we used phage concentrations of 10^8^–10^9^ PFU/mL, aligning with a commonly reported therapeutic range of 10^7^–10^9^ PFU/mL for clinical phage therapy, which varies based on the infection site, administration route, and the patient’s condition (Rodriguez et al., 2022). This concentration range was selected to reflect potential clinical situations where high doses of phages are applied locally and to evaluate the immune responses that might arise under such conditions. However, given that the applied concentrations represent the upper physiological range, this study should be interpreted as an in vitro stress-test designed to reveal potential immune activation under maximal exposure conditions.

**Fig. 1:**
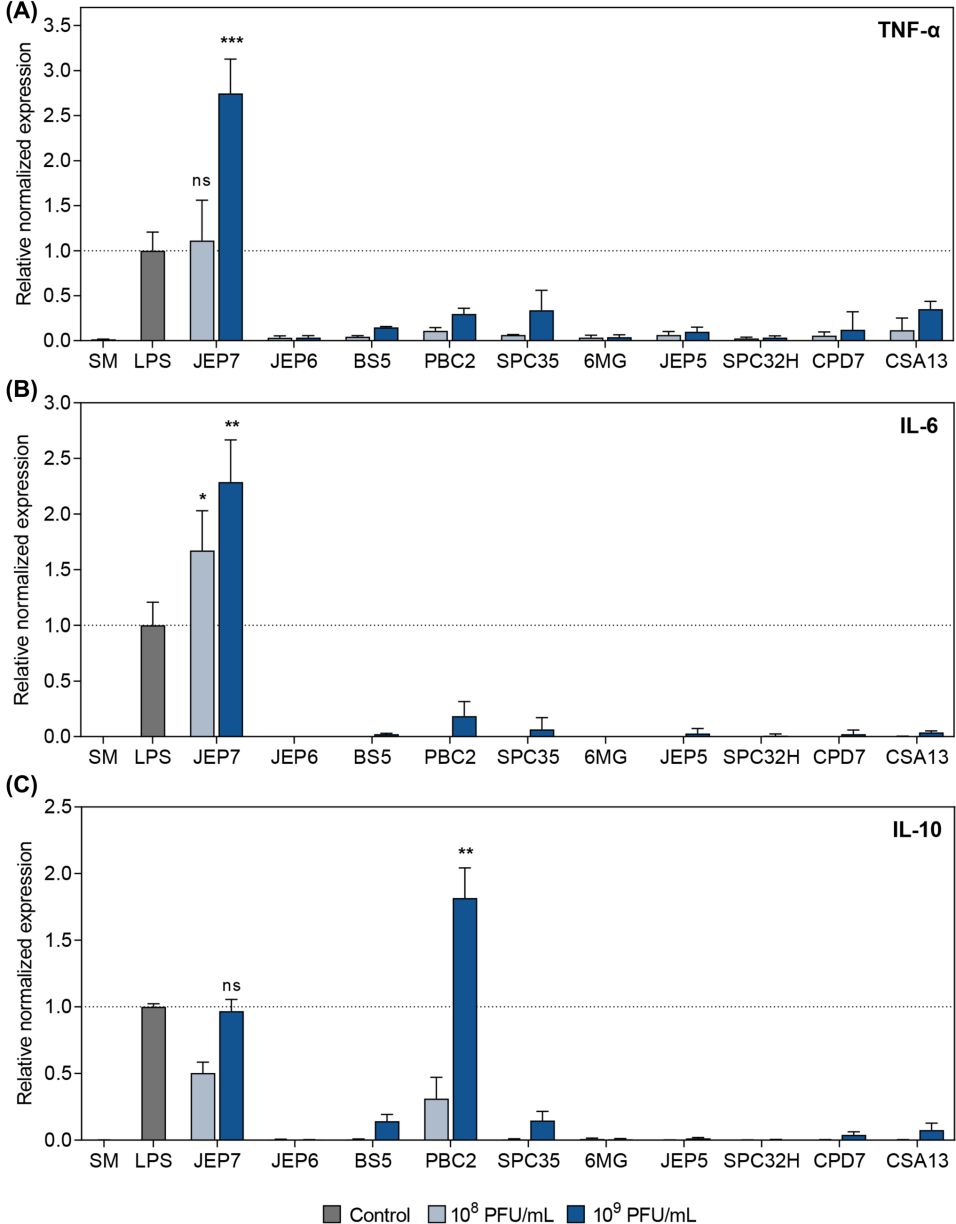
JEP7 and PBC2 induced mRNA expression of inflammatory cytokines in macrophages. RAW 264.7 cells were infected with ten different phages at the concentrations of 10^8^ or 10^9^ PFU/mL. The relative mRNA expression levels of (**A**) TNF-α, (**B**) IL-6, and (**C**) IL-10 were quantified using qRT-PCR. SM buffer-treated cells (SM) and LPS-stimulated cells (LPS) served as the negative and positive controls, respectively. The dotted lines indicate the average levels of mRNA expression induced by the LPS-stimulated cells. Error bars represent the mean ± standard deviation (SD) from biologically independent experiments (control, n = 4; phage-treated groups, n = 3). Asterisks indicate significant differences compared to the positive control (LPS) (Unpaired *t*-test with Welch’s correction; *p < 0.05; **p < 0.01; ***p < 0.005; *ns*, not significant)

When each of the ten phages was incubated with RAW 264.7 cells for 24 h (Fig. 1), JEP7 was the only phage to induce significantly higher expression of the pro-inflammatory cytokines TNF-α and IL-6. This response exceeded the levels induced by LPS (≥ 500 EU/mL) even after endotoxin removal (Fig. 1A, B). Numerous studies have reported contradictory findings on whether phages can directly induce cytokine responses in mammalian immune systems. Some studies have suggested that the phage-induced cytokine production might result from residual bacterial components present in insufficiently purified phage preparations (Pennetzdorfer et al., 2023; Van Belleghem et al., 2017a), while others indicate that certain phages might trigger cytokine responses even in the absence of such contaminants, although the precise mechanisms remain unclear and may vary depending on the phages involved (Dufour et al., 2019; Yildizli et al., 2020). To clarify the contribution of phage particles themselves, rigorous purification is essential. JEP7 induced markedly higher levels of TNF-α, IL-6, and IL-10 in RAW 264.7 cells compared to stimulation with a significantly high concentration of LPS (≥ 500 EU/mL), indicating potent immunostimulatory effects intrinsic to the phage particles. Endotoxin was detected at a concentration of 0.338 EU/mL in the purified JEP7 preparation following multiple purification steps (Table S3), a level within the USP safety threshold of 0.5 EU/mL. Thus, the substantial cytokine induction observed is unlikely to be attributable to endotoxin contamination.

In contrast, the other three phages that underwent endotoxin removal (JEP5, JEP6, and 6MG) no longer induced cytokine expression to the levels observed prior to endotoxin removal. This was in line with the remaining six phages, which showed no significant effect. These results indicate that JEP7 may elicit a pro-inflammatory response in RAW 264.7 cells. Notably, both JEP7 and PBC2 stimulated the expression of the anti-inflammatory cytokine IL-10 (Fig. 1C). However, PBC2 induced a markedly higher IL-10 expression compared to LPS, while failing to upregulate TNF-α and IL-6 to the levels observed with either LPS or JEP7 (Fig. 1A, B). Given that IL-10 plays a key role in modulating pro-inflammatory cytokine responses and maintaining immune homeostasis, the IL-10 expression induced by JEP7 may reflect a secondary response to its strong induction of TNF-α and IL-6.

**Fig. 2:**
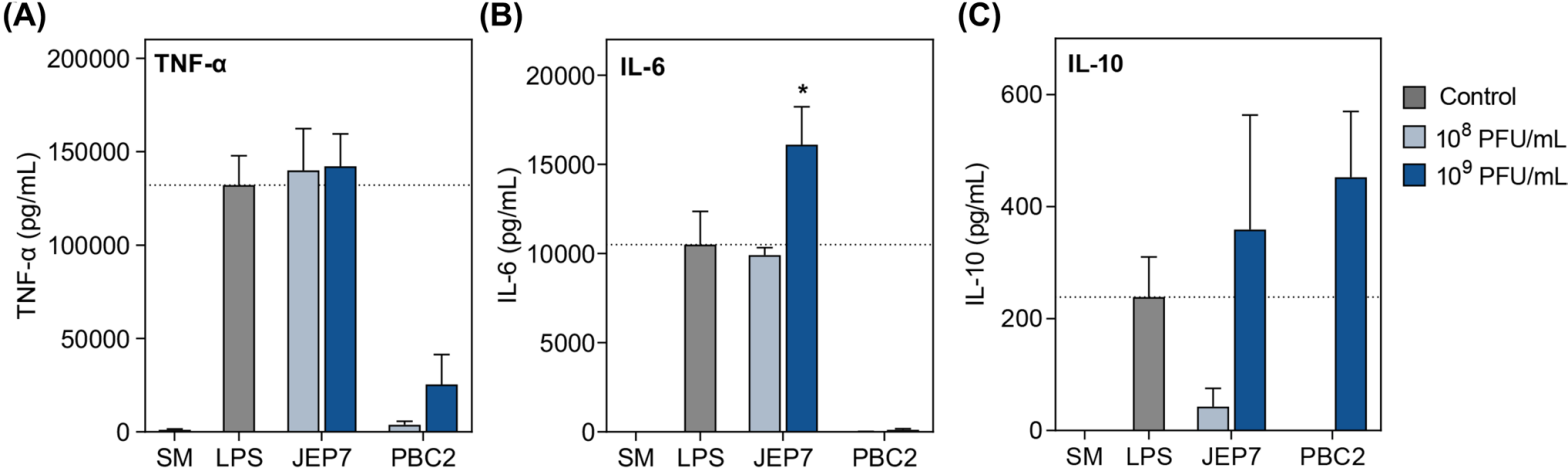
JEP7 and PBC2 induced the production of inflammatory cytokines in macrophages. RAW 264.7 cells were infected with JEP7 or PBC2 at the concentrations of 10^8^ or 10^9^ PFU/mL. The concentrations of (**A**) TNF-α, (**B**) IL-6, and (**C**) IL-10 induced by the phages were measured using ELISA. SM buffer-treated cells (SM) and LPS-stimulated cells (LPS) served as the negative and positive controls, respectively. The dotted lines indicate the average mRNA expression levels for the LPS-stimulated cells. Error bars represent the mean ± standard deviation (SD) from biologically independent experiments (n = 3). Asterisks indicate significant differences compared to the positive control (LPS) (Unpaired *t*-test with Welch’s correction; *p < 0.05)

qRT-PCR results were validated by measuring the levels of TNF-α, IL-6, and IL-10 in RAW 264.7 cells using an enzyme-linked immunosorbent assay (ELISA) following incubation with JEP7 or PBC2 (Fig. 2). JEP7 induced the production of TNF-α and IL-6, while PBC2 promoted IL-10 production to levels comparable to those observed in the LPS-stimulated cells. The secreted cytokine profiles closely mirrored the transcriptional patterns obtained by qRT-PCR, supporting the notion that cytokine production 24 h after phage exposure represents a biologically meaningful response.

Collectively, these results emphasize the distinctive roles of JEP7 and PBC2 in stimulating macrophage responses. JEP7 primarily triggers a pro-inflammatory response while PBC2 predominantly stimulates an anti-inflammatory response, highlighting their contrasting effects on inflammatory response. Although a minor amount of endotoxin (0.338 EU/mL) was detected in the purified JEP7 preparation, this level is below the USP safety threshold (0.5 EU/mL) and far lower than that of the LPS positive control (≥ 500 EU/mL). Therefore, the marked induction of TNF-α and IL-6 observed with JEP7 is most likely attributable to intrinsic immunostimulatory properties of the phage itself rather than residual endotoxin.

### JEP7 and PBC2 also triggered inflammatory cytokine expression in epithelial cells

**Fig. 3:**
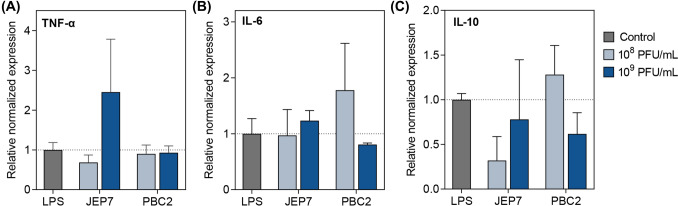
JEP7 and PBC2 also triggered inflammatory cytokine expression in epithelial cells. Caco-2 cells were incubated with JEP7 or PBC2 at the concentrations of 10^8^ or 10^9^ PFU/mL. The relative mRNA expression levels of (**A**) TNF-α, (**B**) IL-6, and (**C**) IL-10 were quantified using qRT-PCR. LPS-stimulated cells (LPS) served as the positive control, with the dotted lines indicating their average mRNA expression levels. Error bars represent the mean ± standard deviation (SD) from biologically independent experiments (n = 3)

Epithelial cells, similar to immune cells, play a crucial role in innate immune responses by synthesizing and secreting inflammatory cytokines, serving as a primary defense line against invading pathogens (Ma et al., 2003). To determine whether JEP7 and PBC2 induce cytokine expression in epithelial cells as observed in macrophages, we measured the expression of TNF-α, IL-6, and IL-10 in Caco-2 cells (Fig. 3). JEP7 induced expression of TNF-α and IL-6 to levels comparable to those observed in LPS-stimulated cells (Fig. 3A, B), indicating that JEP7 predominantly promotes pro-inflammatory cytokine expression in epithelial cells, similar to its effects on macrophages. PBC2 stimulated both IL-10 and IL-6 expression to levels comparable to those induced by LPS (Fig. 3C), demonstrating not only a potential anti-inflammatory role but also additional immunostimulatory effects in epithelial cells (Fig. 3C).

In Caco-2 cells, JEP7 triggered a cytokine expression pattern similar to that observed in RAW 264.7 cells, whereas PBC2 induced not only IL-10 but also TNF-α and IL-6, with IL-6 levels exceeding those induced by LPS (Fig. 3). Considering the distinct roles of macrophages and epithelial cells in inflammation and tissue homeostasis, the differential cytokine responses induced by PBC2 might arise from differences in cytokine regulation shaped by the functional characteristics of each cell type. Supporting this notion, previous studies have shown that anti-inflammatory agents like glucocorticoids regulate cytokine production differentially in macrophages compared with epithelial cells (Vetillard and Schlecht-Louf, 2018). Taken together, these findings highlight how a single phage may exhibit diverse immunostimulatory effects in both immune and non-immune cells.

### Phage internalization did not influence cytokine production in macrophages induced by JEP7 and PBC2

**Fig. 4:**
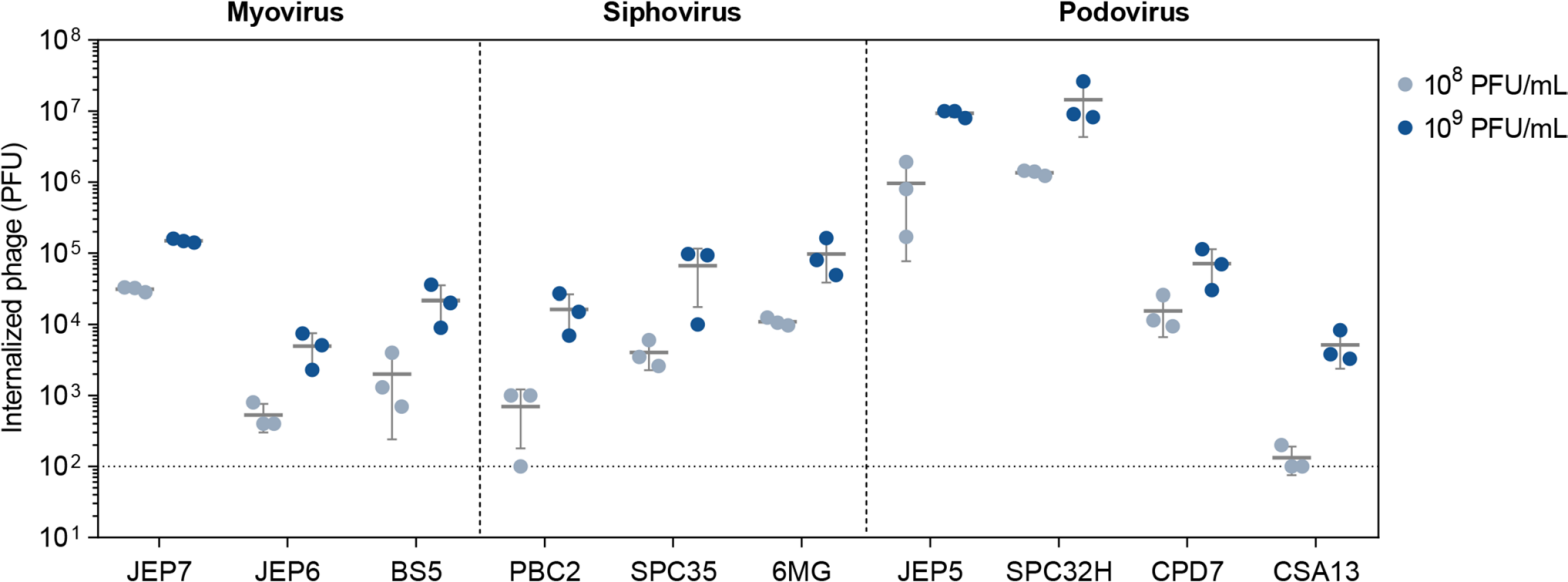
Phage internalization is independent of cytokine production induced by JEP7 and PBC2 in macrophages. Quantification of internalized phages in RAW 264.7 cells. RAW 264.7 cells were infected with each of the ten phages for 24 h, after which the number of intracellular phages was quantified as PFU. Based on their morphology, the phages are grouped into Myovirus-like (JEP7, JEP6, BS5), Siphovirus-like (PBC2, SPC35, 6MG), and Podovirus-like phages (JEP5, SPC32H, CPD7, CSA13). The dotted lines indicate the detection limit of the assays. Error bars represent the mean ± standard deviation (SD) from biologically independent experiments (n = 3)

To determine whether the cytokine responses induced by JEP7 and PBC2 are associated with phage internalization into mammalian cells, we assessed the internalization rates of ten phages into RAW 264.7 cells by quantifying the PFUs of internalized phages (Fig. 4). Since phage size and morphology are the potential factors influencing the internalization rate (Bichet et al., 2021), the phages were classified into three morphological groups: Myoviruses (JEP7, JEP6, and BS5), Siphoviruses (PBC2, SPC35, and 6MG), and Podoviruses (JEP5, SPC32H, CPD7, and CSA13). Within the Myovirus group, JEP7 exhibited a significantly higher internalization rate compared to JEP6 and BS5 at both concentrations of 10^8^ and 10^9^ PFU/mL (*p* < 0.0001, one-way ANOVA with Dunnett’s post hoc test). In contrast, within the Siphovirus group, PBC2 showed a significantly lower internalization rate compared to SPC35 and 6MG at 10^8^ PFU/mL (*p* = 0.04 and *p* = 0.0002, respectively, one-way ANOVA with Dunnett’s post hoc test), whereas no significant differences were observed at 10^9^ PFU/mL (*p* = 0.1603, one-way ANOVA). Although both JEP7 and PBC2 strongly induced cytokine production in macrophages (Fig. 1), they exhibited markedly different internalization rates. This discrepancy is further highlighted by SPC32H (Podovirus), which exhibited the highest internalization rate among all the phages tested but did not affect cytokine production. Collectively, these findings indicate that phage internalization alone is not sufficient to drive cytokine production in macrophages, suggesting that additional phage-specific factors contribute to immune activation.

Due to its strong ability to induce pro-inflammatory cytokines, JEP7 may potentially activate innate immune sensing pathways such as TLR4, irrespective of endotoxin contamination. In addition, recent studies suggest that phage capsids can disassemble within endo-lysosomes following internalization, leading to the release of dsDNA, which could be recognized by the TLR9 or cGAS–STING pathway (Gogokhia et al., 2019). This process may contribute to the production of TNF-α and IL-6. This mechanism is proposed as a possible explanation for the pro-inflammatory responses elicited by JEP7; however, it remains a speculative hypothesis based on previous studies and warrants rigorous experimental investigation to define the molecular basis of this immune activation.

PBC2 promoted the production of the anti-inflammatory cytokine IL-10, which inhibits pro-inflammatory cytokine production (Fiorentino et al., 1991). While previous studies have reported that *Pseudomonas aeruginosa* phage Pf4 can activate TLR3 to induce anti-inflammatory cytokines via phage-associated RNA or outer membrane vesicles (OMVs) (Pennetzdorfer et al., 2023; Sweere et al., 2019), this mechanism does not account for the property of PBC2 to induce anti-inflammatory cytokine. Unlike Pf4, *B. cereus* phage PBC2 is not associated with OMVs, as its host bacterium lacks an outer membrane. In addition, TLR3 signaling typically requires the recognition of double-stranded (ds) RNA within endosomes (Matsumoto et al., 2014). However, PBC2, a dsDNA phage, shows the lowest internalization rate among the tested phages (Fig. 4). This low internalization rate implies restricted access to endosomal TLR3, potentially limiting its ability to engage with this specific immune receptor. Furthermore, PBC2 induced strong IL-10 production in macrophages without elevating TNF-α or IL-6 (Figs. 1 and 2), diverging from the canonical TLR3 signaling that often activates both pro- and anti-inflammatory cytokines (Hsieh et al., 2025). This suggests that IL-10 induction by PBC2 might rely on alternative pathways, such as TLR9 sensing phage DNA (Carroll-Portillo and Lin, 2019; Gogokhia et al., 2019) or TLR2-mediated recognition of viral structural proteins (Lin et al., 2020), potentially explaining the ability of PBC2 to induce anti-inflammatory cytokine IL-10.

### Cytotoxicity of phages is not associated with cytokine production in macrophages induced by JEP7 and PBC2

**Fig. 5:**
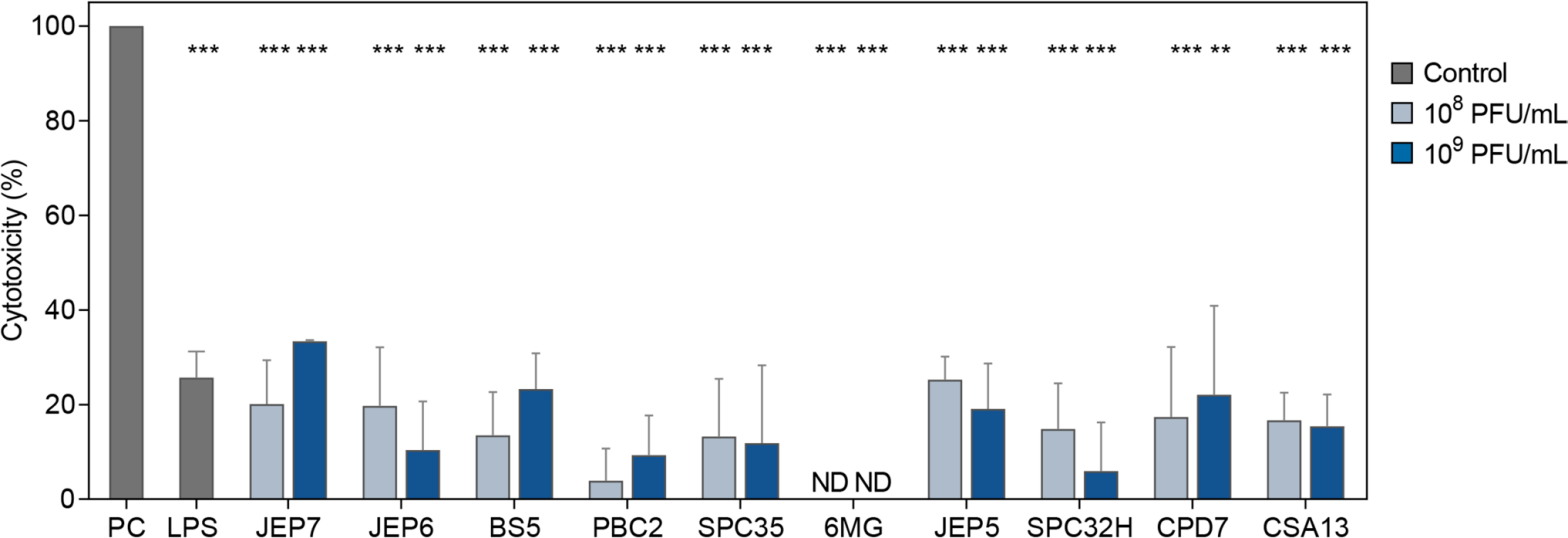
Cytotoxicity of phages is not associated with cytokine production induced by JEP7 and PBC2 in macrophages. Cytotoxicity of phages against RAW 264.7 cells. Cell cytotoxicity was determined by measuring LDH release from RAW 264.7 cells incubated with each of the phages or LPS (1 μg/mL) for 24 h. Cells lysed with 2% Triton X-100 for 15 min to release total LDH (100%) served as the positive control (PC). Error bars represent the mean ± standard deviation (SD) from three biologically independent experiments (n = 3). Asterisks indicate significant differences to the positive control (2% Triton X-100) (Unpaired *t*-test with Welch’s correction; **p < 0.01; ***p < 0.005). ND, not detected

Another possibility is that the cytokine responses in macrophages induced by JEP7 and PBC2 might result from phage-induced cytotoxicity. To investigate this hypothesis, we compared the cytotoxicity of JEP7 and PBC2 against RAW 264.7 cells with that of the other phages that did not elicit cytokine production using the lactate dehydrogenase release assay (Fig. 5). To establish a reference for the highly cytokine-inducing agent, we also assessed the cytotoxicity of LPS, a well-known and potent stimulator of cytokine responses. Among the ten phages tested, varying degrees of cytotoxicity were observed for all except 6MG, which showed no detectable effect. However, all phages and LPS demonstrated significantly lower cytotoxicity compared to the positive control (2% Triton X-100). Notably, LPS exhibited cytotoxicity levels comparable to those of JEP7 and JEP5. Although the phages displayed comparable levels of cytotoxicity, JEP7 was the only one that induced cytokine production in RAW 264.7 cells (Fig. 1), indicating that cytokine responses induced by phages are not directly associated with their cytotoxicity. This interpretation is further supported by PBC2, which induced even stronger IL-10 expression than LPS at 10⁹ PFU/mL (Fig. 1C), while exhibiting significantly lower cytotoxicity at both 10^8^ PFU/mL (*p* = 0.0125) and 10^9^ PFU/mL (*p* = 0.0473). We also evaluated phage cytotoxicity against Caco-2 cells and observed that all phages tested showed only minimal cytotoxicity, indicating that these phages do not induce appreciable cytotoxic effects (Fig. S1). In summary, these findings suggest that the cytokine responses induced by JEP7 and PBC2 are not a consequence of cytotoxicity but are more likely mediated through the activation of distinct intracellular signaling pathways in response to specific phage components.

### Protein glycosylation was not observed in any of the phages tested

**Fig. 6:**
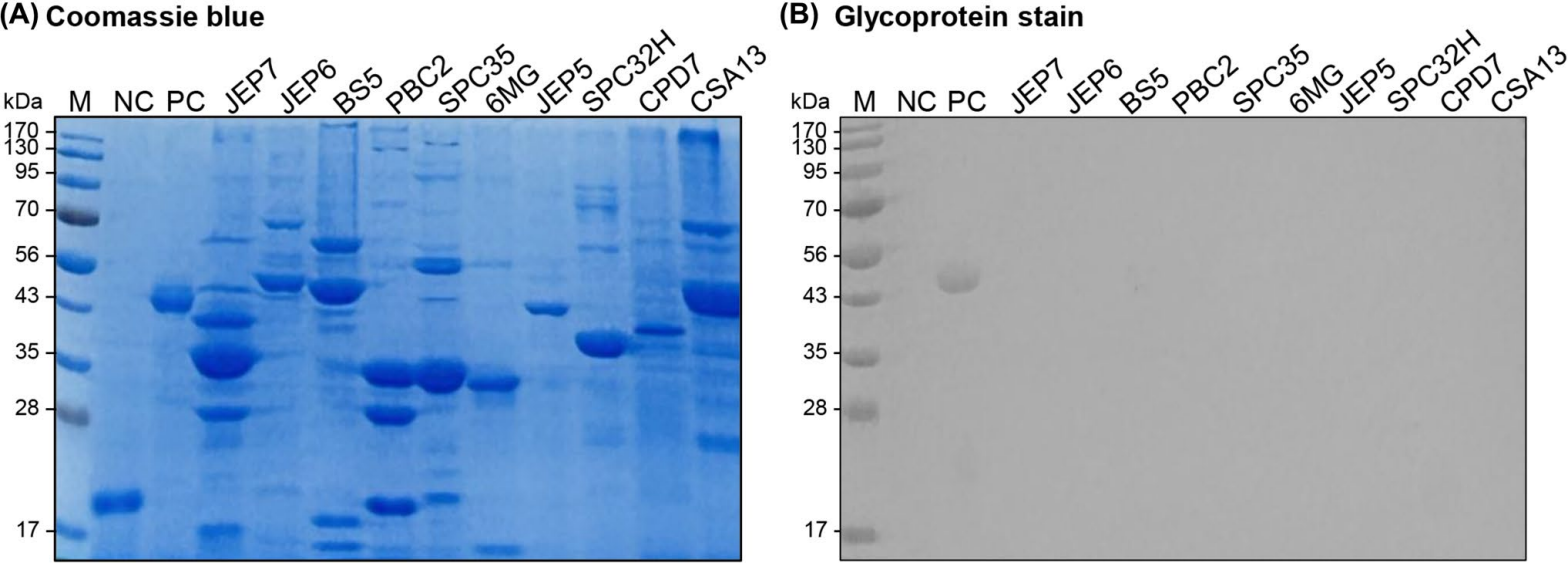
Virion glycosylation of phages is not the primary driver of cytokine production induced by JEP7 and PBC2. SDS-PAGE analysis of surface proteins from ten phages, stained with (**A**) Coomassie blue or (**B**) Glycostain. M, Molecular weight markers; NC, Soybean trypsin inhibitor (18 kDa negative control for glycoprotein staining); PC, Horseradish peroxidase (44 kDa positive control for glycoprotein staining)

Virion glycosylation has been associated with changes in mammalian immune responses, potentially by affecting the recognition of viral surface structures by the immune system (Freeman et al., 2023). To investigate whether such modifications might contribute to the cytokine production induced by JEP7 and PBC2, we analyzed the surface glycoprotein profiles of ten phages (Fig. 6). All phages revealed several abundant protein bands, likely structural components such as capsid or tail subunits, along with other less abundant bands in the Coomassie staining (Fig. 6A). However, no visible glycoprotein bands were observed in any of the analyzed phages using a glycoprotein stain (Fig. 6B), indicating that glycoprotein concentrations, if present, were below the assay’s limit of detection. Although PBC2 carries a gene (*PBC2_047*) annotated as a putative methyltransferase, which may be involved in protein glycosylation (Freeman et al., 2023), no evidence of glycosylated proteins was observed in this phage. Hence, the cytokine production stimulated by JEP7 and PBC2 is unlikely to be driven by glycosylation of phage surface proteins.

In Freeman et al. (Freeman et al., 2023), glycoprotein staining showed strong signals when structural proteins were detected at levels comparable to those observed in our study. In contrast, despite the presence of abundant structural proteins, no glycoprotein signals were detected in our analysis. This marked difference strongly suggests that glycosylation is genuinely absent in the phages analyzed, making additional high-sensitivity analyses unnecessary for further validation.

In conclusion, we demonstrate that certain phages, including JEP7 and PBC2, can induce distinct cytokine responses in both macrophages and epithelial cells. To investigate factors that might explain the cytokine-inducing ability of JEP7 and PBC2, we compared internalization rates, cytotoxicity, and virion glycosylation with those of phages that did not affect cytokine production. No significant differences were observed, suggesting that these characteristics are unlikely to be the primary drivers of cytokine production by phages. This comparative analysis underscores the intricate nature of phage–host immune interactions and highlights the importance of uncovering additional molecular determinants.

## Supplementary Information

Below is the link to the electronic supplementary material.

Supplementary file1 (DOCX 224 KB)
