# Supplementary material for: Bacteriophages JEP7 and PBC2, which target foodborne pathogens, elicit cytokine responses in mammalian cells
Source: Food Sci Biotechnol. 2025 Nov 26;35(3):689–99. doi: 10.1007/s10068-025-02042-3 (PMC12894461; doi:10.1007/s10068-025-02042-3)
Supplement: Supplementary file 1 — Supplementary file1 (DOCX 224 KB) [file 10068_2025_2042_MOESM1_ESM.docx]

Supplementary information for:

**Bacteriophages JEP7 and PBC2, Which Target Foodborne Pathogens, Elicit Cytokine Responses in Mammalian Cells**

**Yewon Jung^1,2^†, Jinshil Kim^1,2,3,4^†**‡**, Ju-Hoon Lee^1,2,3^, Sangryeol Ryu^1,2,3^#**

^1^Department of Food and Animal Biotechnology, Research Institute of Agriculture and Life Sciences, Seoul National University, Seoul,08826, Republic of Korea.

^2^Department of Agricultural Biotechnology, Seoul National University, Seoul, 08826, Republic of Korea.

^3^Center for Food Bioconvergence, Seoul National University, Seoul, 08826, Republic of Korea

^4^Department of Food Science & Biotechnology, and Carbohydrate Bioproduct Research Center, Sejong University, Seoul, 05006, Republic of Korea.

†These authors contributed equally to this work and share first authorship.

‡Present address: Gene Expression and Regulation Section, Laboratory of Biochemistry and Genetics, National Institute of Diabetes and Digestive and Kidney Diseases, National Institutes of Health, Bethesda, MD, 20892, USA.

**For correspondence**

#E-mail: sangryu@snu.ac.kr; Tel: +82-2-880-4856; Fax: +82-2-873-5095

**This docx file includes:**

1 Supplementary figure, 3 Supplementary tables

**
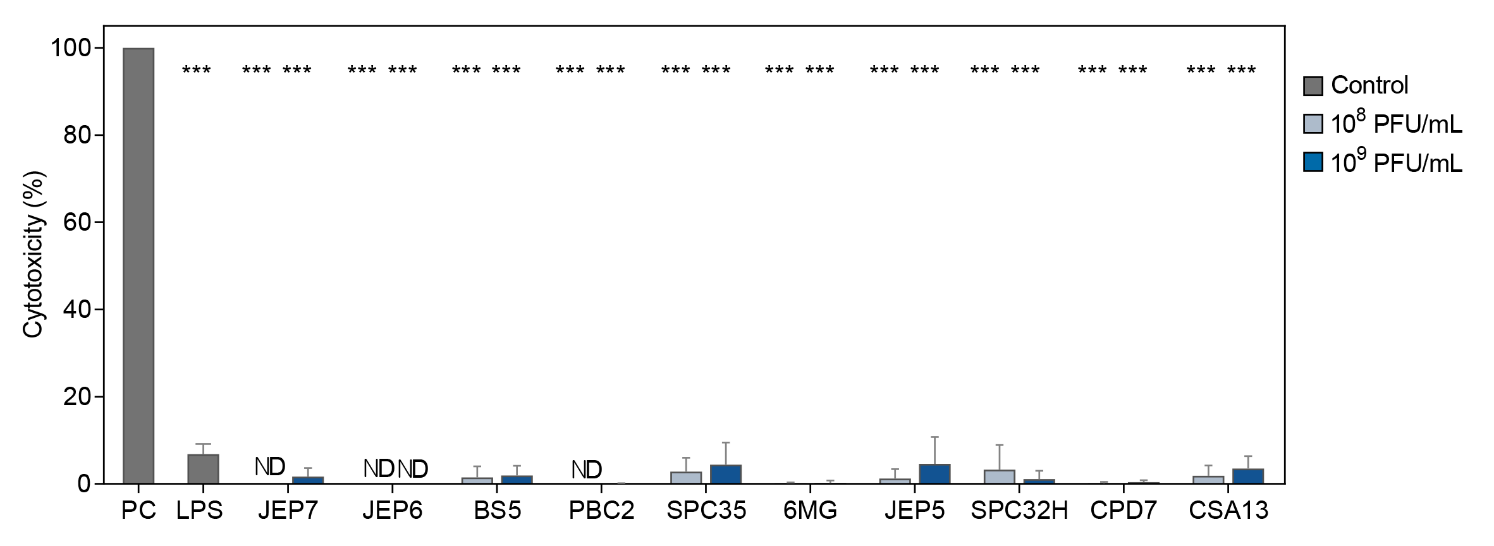
**

**Fig. S1** Cytotoxicity of phages against Caco-2 cells. Cell cytotoxicity was determined by measuring LDH release from Caco-2 cells incubated with each of the phages or LPS (1 μg/mL) for 24 h. Cells lysed with 2% Triton X-100 for 15 min to release total LDH (100 %) served as the positive control (PC). Asterisks indicate significant differences to the positive control (2% Triton X-100) (Student’s *t*-test; ****p* < 0.005). ND, not detected

**Table S1** All phage strains used in this study

| **Morphotype** | **Phage** | **Propagation host** | **Life**  **cycle** | **Size (nm)** | | **GenBank**  **accession** | **Reference** |
| --- | --- | --- | --- | --- | --- | --- | --- |
|  |  |  |  | **Head** | **Tail** |  |  |
| Myovirus | JEP7 | *Escherichia coli* (E45) | Virulent | 103.9 | 95.2 | MT764207 | ([Kim et al., 2021a](#_ENREF_5); [Kim et al., 2021b](#_ENREF_6)) |
|  | JEP6 | *Escherichia coli* (E79) | Virulent | 109.1 | 110.3 | MT764206 | ([Kim et al., 2021a](#_ENREF_5); [Kim et al., 2021b](#_ENREF_6)) |
|  | BS5 | *Bacillus thuringiensis*  (ATCC 10792) | Unidentified | 72.1 | 169.8 | Unpublished | Lab collection |
| Siphovirus | PBC2 | *Bacillus cereus*  (ATCC 13061) | Temperate | 80 | 500 | KT070867 | ([Kong et al., 2019](#_ENREF_9)) |
|  | SPC35 | *Salmonella enterica* Typhimurium (SL1344) | Virulent | 70 | 154 | HQ406778 | ([Kim and Ryu, 2011](#_ENREF_7)) |
|  | 6MG | *Escherichia coli* (MG1655) | Temperate | 70.7 | 187.5 | Unpublished | Lab  collection |
| Podovirus | JEP5 | *Escherichia coli* (E65) | Unidentified | 72.2 | 16.6 | Unpublished | ([Kim et al., 2021b](#_ENREF_6)) |
|  | CSA13 | *Staphylococcus aureus*  (Isolate FMB-1) | Virulent | 40 | Short, non-contractile | MH107118 | ([Cha et al., 2019](#_ENREF_1)) |
|  | CPD7 | *Clostridium perfringens*  (Isolate 2722) | Virulent | 43 | Short, non-contractile | MK017820 | Lab collection |
|  | SPC32H | *Salmonella enterica* Typhimurium (LT2(c)) | Temperate | 62.3 | 15.4 | KC911856 | ([Kim and Ryu, 2013](#_ENREF_8)) |

**Table S2** Primers used in this study

| **Target** | **Primer** | **Sequence (5'-3')** | **Amplicon**  **Size (bp)** | **Reference** |
| --- | --- | --- | --- | --- |
| **RAW 264.7** | | | | |
| GAPDH | GAPDH-F | GAAGGTCGGTGTGAACGGAT | 193 bp | ([Dai et al., 2017](#_ENREF_2)) |
|  | GAPDH-R | GACAAGCTTCCCATTCTCGG |  |  |
| TNF-α | TNF-α-F | GAAGAGGCACTCCCCCAAAA | 188 bp | ([Gabande-Rodriguez et al., 2019](#_ENREF_4)) |
|  | TNF-α-R | TGGGCCATAGAACTGATGAGA |  |  |
| IL-6 | IL-6-F | GTACTCCAGAAGACCAGAGG | 308 bp | ([Yang et al., 2019](#_ENREF_12)) |
|  | IL-6-R | TGCTGGTGACAACCACGGCC |  |  |
| IL-10 | IL-10-F | CTTACTGACTGGCATGAGGAT | 183 bp | This study |
|  | IL-10-R | TGCATTAAGGAGTCGGTTAGC |  |  |
| **Caco-2** | | | | |
| GAPDH | GAPDH-F | GGAAGGTGAAGGTCGGAGTC | 184 bp | ([Dey and Bradbury, 2017](#_ENREF_3)) |
|  | GAPDH-R | TCAGCCTTGACGGTGCCAT |  |  |
| TNF-α | TNF-α-F | GGGACCTCTCTCTAATCAGC | 182 bp | This study |
|  | TNF-α-R | CACCAGCTGGTTATCTCTCAG |  |  |
| IL-6 | IL-6-F | CCGGGAACGAAAGAGAAGCT | 192 bp | ([Miyahara et al., 2013](#_ENREF_10)) |
|  | IL-6-R | AGAGGTGAGTGGCTGTCTGT |  |  |
| IL-10 | IL-10-F | GACTTTAAGGGTTACCTGGGTTG | 112 bp | ([Wang et al., 2018](#_ENREF_11)) |
|  | IL-10-R | TCACATGCGCCTTGATGTCTG |  |  |

**Table S3** Endotoxin concentrations of phage preparations after endotoxin removal

| **Phage** | **Endotoxin concentrations (EU/mL)^a^** | |
| --- | --- | --- |
|  | **Before^b^** | **After^c^** |
| JEP5 | 957.8 | 0.120 |
| JEP6 | 28.5 | 0.007 |
| JEP7 | 927.9 | 0.338 |
| 6MG | 12.5 | 0.009 |

^a^ Endotoxin concentration measured at a phage concentration of 10^9^ PFU/mL; EU, Endotoxin unit.

^b^ Endotoxin concentrations of the phage preparations before endotoxin removal.

^c^ Endotoxin concentrations of the phage preparations after endotoxin removal.

**REFERENCES**

Cha Y, Chun J, Son B, Ryu S. Characterization and genome analysis of *Staphylococcus aureus* podovirus CSA13 and its anti-biofilm capacity. Viruses 11: 54 (2019)

Dai H, Goto Y-i, Itoh M. Insulin-like growth factor binding protein-3 deficiency leads to behavior impairment with monoaminergic and synaptic dysfunction. Am. J. Pathol. 187: 390-400 (2017)

Dey I, Bradbury NA. Activation of TPA-response element present in human Lemur Tyrosine Kinase 2 (lmtk2) gene increases its expression. Biochem. Biophys. Rep. 12: 140-150 (2017)

Gabande-Rodriguez E, Perez-Canamas A, Soto-Huelin B, Mitroi DN, Sanchez-Redondo S, Martinez-Saez E, Venero C, Peinado H, Ledesma MD. Lipid-induced lysosomal damage after demyelination corrupts microglia protective function in lysosomal storage disorders. EMBO J 38 (2019)

Kim J, Hur JI, Ryu S, Jeon B. Bacteriophage-mediated modulation of bacterial competition during selective enrichment of *Campylobacter*. Microbiol. Spectr. 9 (2021a)

Kim J, Park H, Ryu S, Jeon B. Inhibition of antimicrobial-resistant *Escherichia coli* using a broad host range phage cocktail targeting various bacterial phylogenetic groups. Front. Microbiol. 12: 699630 (2021b)

Kim M, Ryu S. Characterization of a T5-like coliphage, SPC35, and differential development of resistance to SPC35 in *Salmonella enterica* serovar Typhimurium and *Escherichia coli*. Appl. Environ. Microbiol. 77: 2042-2050 (2011)

Kim M, Ryu S. Antirepression system associated with the life cycle switch in the temperate *Podoviridae* phage SPC32H. J. Virol. 87: 11775-11786 (2013)

Kong M, Na H, Ha N-C, Ryu S. LysPBC2, a novel endolysin harboring a *Bacillus cereus* spore binding domain. Appl. Environ. Microbiol. 85: e02462-18 (2019)

Miyahara T, Runge S, Chatterjee A, Chen M, Mottola G, Fitzgerald JM, Serhan CN, Conte MS. D-series resolvin attenuates vascular smooth muscle cell activation and neointimal hyperplasia following vascular injury. FASEB J. 27: 2220 (2013)

Wang X, Luo G, Zhang K, Cao J, Huang C, Jiang T, Liu B, Su L, Qiu Z. Hypoxic tumor-derived exosomal miR-301a mediates M2 macrophage polarization via PTEN/PI3Kγ to promote pancreatic cancer metastasis. Cancer Res. 78: 4586-4598 (2018)

Yang SJ, Lee JE, Lim SM, Kim YJ, Lee NK, Paik HD. Antioxidant and immune-enhancing effects of probiotic Lactobacillus plantarum 200655 isolated from kimchi. Food Sci Biotechnol 28: 491-499 (2019)
